# Diet quality, food security and traditional food intake of pregnant and breastfeeding women, and children 6 months to 5 years, living in eight remote Australian Aboriginal and Torres Strait Islander communities

**DOI:** 10.1186/s12889-025-22815-z

**Published:** 2025-05-01

**Authors:** Emma Tonkin, Mark D. Chatfield, Julie Brimblecombe, Sue Kleve, Ellie Chan, Caroline Deen, Clare Brown, Emma Stubbs, Sue Booth, Kani Thompson, Jenna Pauli, Dympna Leonard, Amanda Lee, Bronwyn Fredericks, Megan Ferguson

**Affiliations:** 1 Department of Nutrition, Dietetics & Food, Monash University, Notting Hill, VIC 3168 Australia; 2 School of Public Health, The University of Queensland, Herston, QLD 4006 Australia; 3 School of Public Health, Menzies School of Health Research, Northern Territory, Casuarina, 0810 Australia; 4 Central Australian Aboriginal Congress, Northern Territory, Alice Springs, 0870 Australia; 5 Apunipima Cape York Health Council, Bungalow, QLD 4870 Australia; 6 College of Medicine and Public Health, Flinders University, Adelaide, South Australia 5000 Australia; 7 School of Population Health, Curtin University, Bentley, WA 6012 Australia; 8 Australian Institute of Tropical Health and Medicine, James Cook University, Cairns, QLD 4870 Australia; 9 Office of the Deputy-Vice-Chancellor, The University of Queensland, Herston, QLD 4006 Australia; 10 Menzies School of Health Research, Northern Territory, Casuarina, 0810 Australia

**Keywords:** Indigenous, First Nations, Food security, Diet, Public health, Food, Australia

## Abstract

**Background:**

Maternal and early childhood nutrition is foundational in setting the course for lifetime metabolic and disease outcomes. Food security influences the achievement of optimal diets; however, little is known about how traditional food intake may influence this dynamic for Aboriginal and Torres Strait Islander people living in remote communities. This study describes diets and food security status of Aboriginal and Torres Strait Islander pregnant and breastfeeding women and children 6 months to 5 years in remote communities in Australia, and explores interactions between diet quality, food security and traditional food consumption.

**Methods:**

Baseline data from a trial testing a discount on healthy foods and drinks were used. Participants from eight communities (four each in coastal Cape York, Queensland and desert Central Australia, Northern Territory) participated in June–September 2021. A validated food frequency questionnaire was used to assess usual intake and calculate a diet quality score. A modified version of the United States Department of Agriculture 18-item Household Food Security Scale Module measured food security status. A model was fitted to explore the interactions between diet quality, food security and traditional food consumption.

**Results:**

Complete dietary data were available for 471 participants from 294 households. Average reported food group intakes of children were similar to recommended patterns, however except for adequate meat intakes those of women were not; mean diet quality scores were 23% higher in children than women (*p* < 0.001). Long-duration breastfeeding was described (36% of > 2–4 years breastfed). High rates of household food insecurity were reported (76%), although rates were lower in Cape York (*p* < 0.001). Reported traditional food intake was higher in Cape York than in Central Australia (*p* < 0.001). For diet quality, a significant three-way interaction between food security status, traditional food frequency and adult/child status was demonstrated (*p* = 0.005).

**Conclusions:**

Remote community families carry out practices that protect and support the diet quality of children despite conditions that challenge food security and optimal diets. The role of traditional food consumption in reducing the impact of food insecurity on diet quality provides further evidence for Aboriginal and Torres Strait Islander food systems to be at the centre of comprehensive efforts to address food insecurity.

**Trial registration:**

This work is part of a trial that has been registered with Australian New Zealand Clinical Trials Registry: ACTRN12621000640808. Trial registration date: 28/05/2021.

## Background

The importance of maternal and early childhood nutrition in setting the lifetime metabolic and disease trajectory, through both direct nutrient and indirect epigenetic effects, is unequivocal [1–3]. Therefore the significance of ensuring access to optimal nutrition for women and children during critical life stages of growth and development, and for the prevention of lifelong chronic disease cannot be overstated [1, 2]. Prior to colonisation, Aboriginal and Torres Strait Islander peoples in Australia had a diverse, health-promoting diet, tightly coupled to season and locality [4], and following customary rules of food sharing, with Elders, pregnant women and children prioritised [5]. Traditional foods include diverse animal (mammal, bird, reptile, insect and marine) and plant species (roots, legumes, seeds, nuts, fruits, nectars, flowers and gums) [4, 6]. However European colonisation has disrupted these traditional food systems, leading to the forced dependence on an inferior diet of highly processed staple foods (flour, sugar) and lacking in fresh foods and variety for people particularly in centralised communities [4, 7, 8]. As a direct consequence of colonisation and its continued power structures, Aboriginal and Torres Strait Islander peoples experience disproportionate rates of food insecurity [9–11], which impact the achievement of dietary patterns consistent with optimal nutrition during the critical period of pregnancy and childhood [12–16]. This is magnified for those living in remote communities (51% of households in remote communities compared with 40% in non-remote areas) [9, 17, 18].

An Aboriginal-defined description of food security is “… when the food of our ancestors is protected and always there for us and our children. It is also when we can easily access and afford the right non-traditional food for a collective healthy and active life. When we are food secure we can provide, share and fulfil our responsibilities, we can choose good food knowing how to make choices and how to prepare and use it” [19]. The many lasting socioeconomic consequences of colonisation for Aboriginal and Torres Strait Islander peoples and other inequities combine to create food supply, access and utilisation challenges that undermine household food security in remote communities [4, 7, 20–22]. Community members report high food costs as the greatest barrier to food security and healthy eating [7, 23], and research substantiates the cost of foods in remote communities is significantly higher than in urban centres [24–26]. In an Australian study exploring barriers to child consumption of fruit and vegetables, remote participants reported more barriers than urban participants, and these barriers related to accessibility, including low incomes, high food prices and pressures to support others [27]. In remote communities food sharing systems exist which reflect complex social systems that are bound by culture, underpinned by Aboriginal and Torres Strait Islander ways of knowing, being and doing, and are based on reciprocity [28, 29]. Overcrowded remote housing conditions can place pressure on these social food sharing systems [7, 22, 23, 27]. Access to nutrient dense traditional foods has been reported as an important strategy for managing household food insecurity in remote locations [7, 30, 31].

In exploring the relationship between food security and overall diet quality, the only Australian study that has been conducted found food insecurity to be associated with lower diet quality scores [32], however Aboriginal and Torres Strait Islander peoples, children, and pregnant and breastfeeding women were intentionally excluded. Internationally results are mixed in adult populations [33–36], and studies in children report either no difference in diet quality with food security status [37], mixed results [38, 39], or the opposite where food insecurity was associated with increased diet diversity in children [40]. Campbell et al. [40] found higher diet quality in food insecure Native Hawaiian, Pacific Islander, and Filipino infants, but did not explore the possibility of increased consumption of traditional foods during times of food scarcity as impacting this relationship. The contribution of traditional foods to positive diet quality outcomes has been particularly demonstrated in Canada [41–47], and in the Northern Territory of Australia consumption of traditional foods has been described as especially important during times of food insecurity [30, 31]. Therefore, increased consumption of traditional foods during times of food insecurity could be hypothesised to change the food security-diet quality relationship, which may go some way to explaining the mixed findings in literature, particularly those demonstrating different outcomes for different ethnic groups with access to traditional foods [33, 35]. Indeed, a Canadian study showed food security was significantly associated with overall diet quality in the general population, but not in First Nations participants [35].

Traditional foods continue to contribute importantly to the diets of some Aboriginal and Torres Strait Islander groups in Australia [4, 17, 30, 48]. Knowledge and skills about traditional foods, and managing the land to support them, is passed intergenerationally in Aboriginal and Torres Strait Islander cultures orally [7]. However the destruction of social connections through forced assimilation, and removal from and dispossession of ancestral lands, has led to a critical disruption of knowledge, identity and culture related to traditional foods for many groups [5, 48, 49]. Aboriginal and Torres Strait Islander cultures are not homogenous, and colonisation has had differing impacts on traditional food systems [48]. In particular, relocation of peoples away from their ancestral lands, therefore forcing dependence on central and immobile points of service delivery (that is, the establishment of missions, now communities) has strongly impacted Aboriginal and Torres Strait Islander groups who travelled large geographical areas in the cultivation and harvesting of traditional foods [5, 6]. Therefore, the impact of colonisation on access to traditional foods is likely to have differing impacts on dietary intake in different geographical regions of Australia.

In addition to describing the consumption of nutritionally dense traditional foods as a way of coping with food insecurity in remote communities, qualitative research exploring the experience of food insecurity with remote Aboriginal and Torres Strait Islander communities has also described the prioritisation of child diets over adult diets when food is scarce [31]. At present, there is no research quantitatively exploring the dynamic between food security, traditional food consumption, and adult/child status, and how they interact to impact diet quality outcomes in women and children in remote Aboriginal and Torres Strait Islander populations. Additionally, there is scant research available describing child diets in exclusively remote communities [17, 18] and none describing maternal diets. This research aims to use dietary intake and food security data collected as part of a co-designed trial of a discount card strategy to reduce the price of healthy foods in remote community stores to explore these research gaps.

## Methods

### Aims

This study aims to:

1. Describe the diet and food security status of Aboriginal and Torres Strait Islander pregnant and breastfeeding women, and children six months to five years, who live in remote communities in two different geographical regions.
2. Explore interactions between diet quality, food security, traditional food consumption and adult/child status.

### Design

This paper reports the dietary and food security baseline data collected as part of the larger, multi-phase, Remote Food Security project [50]. The Remote Food Security project was co-designed with Aboriginal Community Controlled Health Organisations, through longstanding relationships founded in reciprocity. The design process and project governance, including Aboriginal and Torres Strait Islander leadership, has been described elsewhere [50], including how study design and implementation meets best-practice guidelines for research with Aboriginal and Torres Strait Islander peoples [51]. Briefly, in response to a community call to action, the project assessed the impact of a 30% discount on healthy foods and drinks in remote community stores on the diet quality of pregnant and breastfeeding women, and children, using a controlled before-and-after design [50]. The project also explored community-led solutions to improving food security in remote communities more broadly across multiple research phases, the details of which are reported elsewhere [29, 31, 50].

Approval for the research was granted by the Research Governance Committee of Apunipima Cape York Health Council and Central Australian Aboriginal Congress Board. Ethics approval was granted from the University of Queensland (2020/HE000636) and the Central Australian Human Research Ethics Committees (CA-203701. Informed consent was provided by each participant after study procedures had been explained with the use of a participant information sheet.

### Setting and participants

The trial was conducted in eight remote communities, four each in Central Australia in the Northern Territory and Cape York in Queensland. The process for selecting communities is described elsewhere [50]. The distance from the nearest town for the participating communities ranges from approximately 100-1000 km, with six being 300 km or more. The estimated number of eligible people living within each community ranged from 50–125. The four Central Australian communities are desert communities, located in the arid centre of Australia. The four Cape York communities can be described as coastal communities, all situated near the coast in the tropical north of Australia.

All Aboriginal and/or Torres Strait Islander pregnant and breastfeeding women, and parents/primary carers of young children aged six months to five years (hereafter referred to as 'children') were eligible to participate. Dietary data were collected for a maximum of three participants per household, the upper limit thought to be feasible to ask of one adult (e.g., a pregnant or breastfeeding woman could provide dietary data for herself, as well as two children). Lists of eligible community members were provided by community health services and family centres, with community researchers then integral in locating potential participants to invite. Recruitment was primarily conducted at participants’ homes, at the community health clinics or family centres.

### Data collection

Data were collected during interviews with women, or the carers of child participants, in June–September 2021, at any location the participant chose. Interviews were conducted by researchers (including Aboriginal and Torres Strait Islander team members) usually alongside local community researchers. Research team members were trained in the use of the data collection tools by experienced research dietitians. All data were collected via online surveys (Qualtrics International, USA) on iPads, with researchers most commonly reading questions and entering participants’ verbal responses into the survey, but occasionally participants entered their own responses.

Demographic and food preparation hardware (for example oven, refrigerator) and equipment data were collected via the registration survey. The Menzies Remote Short-Item Dietary Assessment Tool (MRSDAT), a 32-item food frequency questionnaire, developed and validated for remote Aboriginal populations, was used to collect most of the dietary intake data [52]. Breastfeeding status was ascertained using a single MRSDAT question asking if the child was breastfed. This measured only current breastfeeding, and did not reflect if the child had ever been breastfed. Similarly, frequency of traditional food consumption was asked as a single question in the MRSDAT for all participants (‘How many times per week do you/does your child usually eat traditional food (wild harvested native fruits, berries, nuts, other plants, and animals like fish, kangaroo, goose, goanna)?’), with response options and coding as follows: ‘none’ = 0, ‘less than 1 time’ = 2, '1–2 times’ = 4, ‘3–4 times’ = 6, ‘5–6 times’ = 8 and ‘everyday’ = 10. Additional information about types of foods commonly consumed (including traditional foods) was collected using a standard 3-pass 24-h recall method [53] with a sub-sample of participants from each community (data recorded on paper and entered into a custom-developed Access database). Minimum Meal Frequency (MMF) for children under two years was determined following the infant and young child indicator outlined by the World Health Organisation [54], with relevant questions incorporated at the end of the MRSDAT questionnaire. A modified version of the United States Department of Agriculture 18-item Household Food Security Scale Module [55], previously used in Australian research [11, 56], was used to measure participants’ food security status.

Surveys containing the demographic and food security data were completed once for each participating household, while the MRSDAT was completed for each individual eligible participant within each household. When multiple MRSDATs were required, they were completed either concurrently (for example, seeking a response to question 1 for each eligible participant) or sequentially (completing the full MRSDAT for the first participant, then moving on to the next) according to participant or data collector preference.

### Analysis

Demographic, dietary intake and food security data are reported with descriptive statistics. To avoid re-identification of individuals or disclosure of specific populations, for variables considered sensitive and identifying cell counts of less than five participants are not reported (instead noted as ‘n/a’). Intakes of all major food groups were calculated using the MRSDAT; five items estimate consumption of fruits and vegetables, four dairy foods, two breads and cereals, four meats and alternatives, five discretionary foods and two sugar-sweetened beverages (SSBs). Estimated intakes are compared to the Australian Dietary Guideline dietary patterns (ADG) [57]. Due to small cell counts, all dietary intakes cannot be presented as proportions of participants meeting recommended intakes, but these have been reported in text where possible.

The MRSDAT was also used to calculate a Dietary Guideline Index (DGI) score [58] based on a diet quality scoring system originally developed for both adults (DGI) and children (DGI-CA) [59–62]. The scoring system enables a numerical comparison of the measured diets with the intake and serving amounts recommended by the ADGs [57]; that is, it compares reported intakes with the recommended intakes for that age and life stage, and provides a score based on how closely the reported and recommended diets align. Detail about the calculation of DGI (also referred to as ‘diet quality’) scores from the MRSDAT can be found elsewhere [58]. Briefly, individual indicator scores for vegetables (score out of 10 [/10]), fruit (/10), grains (/5), dairy (/10), meat (/10), discretionary foods (/20) and SSBs (/5) are calculated based on intakes, with further indicator scores reflecting the recommendations for dietary variety (/10), healthy fats (/10), plain water consumption (/5) and whole grain quality (/5). Indicator scoring formulas are age and gender specific to align with the ADG groupings, and therefore reflect the different nutritional needs at different life stages, including breastfeeding and pregnancy. Total DGI and DGI-CA scores are calculated by summing all indicator scores, with a maximum possible score of 90 for children under two years due to the exclusion of the dairy indicator (see [58]), and 100 for all participants > 2 years. Here, reported DGI-CA scores for participants < 2 years were multiplied by 10/9 to create a score out of 100 for comparability. Diet quality (DGI and DGI-CA) scores were used for all statistical comparisons of diet between women and children.

Responses to the food security questions were coded and categorised following the food security severity scoring outlined by Bickel et al. [55], resulting in four categories: high, moderate, low and very low food security. When describing food security status, we chose to group moderate food security as food insecure, consistent with other Australian research with this tool [11]. While we have used the expanded variable with all four categories of food security severity where possible in analyses, where necessary we grouped high and moderate food security together due to the small numbers within the high food security category (see results figures). Comparisons of outcomes by regions and communities were assessed using Chi-square and Kruskal–Wallis tests. Comparison of diet quality between women and children was assessed using T-tests.

The relationships between diet quality, food security, traditional food intake frequency and adult/child status were explored using a model which included fixed effects for food security status, adult/child status (both dichotomous), traditional food intake frequency code (continuous) and all two-way and three-way interactions, and random effects for families. These interactions are presented graphically, using data from the first child enrolled within each family, i.e. excluding 48 siblings. As DGI-CA and DGI scores were used, which account for the differences in intake required at each age and life stage, children could be grouped into a single group and a dichotomous adult/child variable was used, and the term ‘children’ is therefore used to describe all children aged 6 months to five years included in the model.

## Results

Participant and demographic data can be found in Table 1. Demographic and food security surveys were completed for 294 households, while complete MRSDATs were available for 471 participants (99%), with additional 24-h recall data for 54 participants. In Cape York there were fewer persons per household overnight (*p* < 0.001), and more primary carers had completed higher levels of education (*p* < 0.001), but there were no other differences between the regions.

**Table 1:** Participant and demographic data, by region

	**Cape York**	**Central Australia**
**Participants**	***n* = 256**	***n* = 221**
Women	82 (32%)	81 (37%)
Pregnant	17 (7%)	6 (3%)
Breastfeeding	65 (25%)	75 (34%)
Children	174 (68%)	140 (63%)
6–12 months	16 (6%)	13 (6%)
> 12 to 24 months	44 (17%)	35 (16%)
> 2 to < 5 years	114 (45%)	92 (42%)
**Household/primary carer demographic data**	***n* = 162**	***n* = 132**
Total number of people who stayed at house last night	6.0 (2.5)	7.2 (2.3)
Highest qualifications/level of education completed		
Primary	10 (6%)	24 (18%)
Yr 10 or equivalent	86 (53%)	79 (60%)
Yr 12 or equivalent	35 (22%)	20 (15%)
Trade or university qualification	31(19%)	5 (4%)
Never attended school/don’t know	n/a^a^	n/a^a^
Current employment^b^		
Full time work	17 (10%)	14 (11%)
Part time work	7 (4%)	9 (7%)
Casual work	20 (12%)	15 (11%)
Home duties	132 (81%)	15 (11%)
Not working	26 (16%)	78 (59%)
Study	13 (8%)	0 (0%)
Food preparation hardware and equipment		
Working stove, oven or other cooking facilities	134 (83%)	109 (83%)
Benches in good working order	152 (94%)	130 (98%)
Kitchen cupboards in good working order	138 (85%)	107 (81%)
Working refrigerator	144 (89%)	119 (90%)
Cooking and eating utensils that are in good working order	158 (98%)	124 (95%)

^a^small cell count size

^b^options were not mutually exclusive, therefore columns do not add up to 100%

### Dietary intake

#### Food group intakes compared to the Australian Dietary Guidelines

The median intakes of each food group of the reported diets for children and women are reported in Table 2. Median reported intakes suggest children’s diets were similar to ADG recommended dietary patterns, however, women were not able to consume most food groups in the recommended quantities (Table 2). Most women (64%) and children (including all ages) (71%) however reported meeting recommended meat intakes. Notably, 83% of infants 6–12 months old were reported to meet recommended vegetable intakes, while this declined to 32% of children aged 12- < 24 months and 4% of children aged 2–5 years, and even fewer women. While it was reported that most infants 6–12 months were not consuming SSBs (62%), and many not consuming discretionary foods (35%), on average both children and women had higher reported SSB and discretionary food intakes than recommended; 94% of children 2–5 years and women (combined) reported consuming SSBs at least occasionally, and 85% weekly or more. Reported food group intakes were closer to recommended patterns in Cape York than Central Australia.

**Table 2:** Daily caregiver or self-reported intake compared with the Australian Dietary Guideline (ADG) recommended intakes, by region

	Children < 2 years			Children 2–5 years			Women		
**Food or food group**	**Cape York,* n* = 60**	**Central Aust,* n* = 48**	**ADG serves**^**a**^	**Cape York,* n* = 112**	**Central Aust,* n* = 92**	**ADG serves**^**a**^	**Cape York,* n* = 81**	**Central Aust,* n* = 80**	**ADG serves**^**a**^
	**Median serves (IQR)**			**Median serves (IQR)**			**Median serves (IQR)**		
Vegetable	1.0 (0.7, 2.3)	1.0 (0.6, 1.4)	0.4–3.0	1.2 (0.8, 2.3)	1.0 (0.7, 1.3)	2.5–4.5	1.6 (1.0, 2.4)	1.3 (0.9, 2.1)	5.0–7.5
Fruit	1.0 (0.4, 2.0)	0.5 (0.2, 1.0)	0.07–0.5	2.0 (0.9, 3.0)	1.0 (0.6, 2.0)	1.0–1.5	0.9 (0.4, 2.0)	0.4 (0.2, 1.0)	2.0
Grain	3.0 (3.0, 5.0)	3.0 (1.0, 3.0)	2.5–4.0	5.0 (3.0, 5.0)	3.0 (3.0, 3.0)	4.0	5.0 (5.0, 7.0)	3.0 (3.0, 5.0)	8.5–9.0
Meat	1.2 (0.9, 1.7)	0.9 (0.7, 1.3)	0.5–1.0	1.5 (1.2, 2.0)	1.2 (1.0, 1.6)	1.0–1.5	2.9 (2.1, 3.7)	3.1 (2.3, 3.6)	2.5–3.5
Dairy	2.1 (0.9, 3.4)	1.4 (1.0, 2.4)	1.0–1.5	2.4 (1.4, 3.4)	1.9 (1.3, 2.4)	1.5–2.0	1.9 (1.1, 3.1)	1.3 (1.0, 2.0)	2.5
SSB	0.2 (0.0, 0.4)	0.2 (0.0, 0.4)	0.0	0.4 (0.1, 0.6)	0.4 (0.1, 0.6)	0.0	0.4 (0.1, 1.0)	0.4 (0.1, 1.0)	0.0
Discretionary	1.8 (0.8, 2.7)	2.1 (1.3, 2.5)	0.0	2.5 (2.3, 3.5)	2.8 (2.3, 4.3)	0.0–1.0	2.5 (2.0, 3.5)	3.8 (2.5, 5.0)	0.0–2.5

^a^*ADG* Australian Dietary Guideline recommended daily serves for each age group, for each food or food group. Recommended intakes for children 7–12 months have been adjusted for weight equivalents. Where multiple ADG age groups are clustered within the participant age group categories, a range for the recommended number of serves has been provided; for children, the higher recommended range relates to older children within the group, and the lower younger

#### Diet quality (DGI and DGI-CA scores)

While DGI scores ranged from 23.1 to 90.2 out of 100, small interquartile ranges indicate modest data spread within groups overall (median DGI score [IQR]: children < 2 years, 56 [50, 61]; children 2–5 years, 58 [51, 63]; women, 45 [39, 52]). Reflecting the intake patterns above, average scores were higher for children (M = 56.9, SD = 9.4) than women (M = 46.2, SD = 10.5, *p* < 0.001). Except grain quality intake, all diet quality scores were higher in Cape York than Central Australia, and in particular the higher consumption of seafood supported greater healthy fats indicator scores in Cape York (Table 3).

**Table 3:** DGI/DGI-CA scores, and component indicator scores, by region

	**Cape York**	**Central Aust**
**Indicator (max. score)**	*n* = 253	*n* = 220
DGI score (/100)	56.8 (10.5)^a^	49.1 (10.2)^b^
Vegetable (/10)	4.8 (3.2)	3.8 (2.8)
Fruit (/10)	7.7 (3.2)	6.5 (3.7)^b^
Grain intake (/5)	4.0 (1.1)	3.1 (1.2)
Wholegrain quality (/5)	1.2 (1.6)	1.5 (1.6)
Meat (/10)	9.2 (1.7) ^a^	9.0 (1.8)
Dairy^c^ (/10)	8.1 (2.7)	7.2 (2.9)
Healthy fats (/10)	5.7 (1.9)	3.5 (1.9)
Discretionary (/20)	1.6 (4.4)	0.6 (3.1)
SSB (/5)	2.8 (1.8)	2.7 (1.8)
Dietary variety (/10)	7.2 (1.5)	6.5 (1.3)

^*a*^*n* = 252, ^b^*n* = 219, ^c^dairy indicator scores are not calculated for children < 2 years, therefore sample sizes are Cape York *n* = 193, Central Australia *n* = 172

Commonly consumed foods ranged in quality. Most (91%) participants reported to eat three or more, and 32% reported eating five, different types/colours of vegetables. Typical meat and alternatives group foods eaten included many high-quality sources like beef steak, chicken breast, eggs, kangaroo and fresh fish and seafood, as well as some low-quality meats like bacon, ham, sausages, and chicken nuggets. Importantly given its typical nutrient density, 41% of all participants reported consumption of offal. White breads, damper and rice, and instant noodles were the most commonly consumed grain foods, high-quality and fortified breakfast cereals/oats, pasta and wholegrain breads were also frequently reported. Milk was consumed daily by most participants (79%), and 95% said this was usually plain/unsweetened, while cheese and yoghurt were much less frequently consumed (23% of participants reported daily consumption). Fresh, whole fruits, and fruit juice, were frequently eaten by children, and less commonly by adults.

#### Traditional food intake

**Fig. 1:**
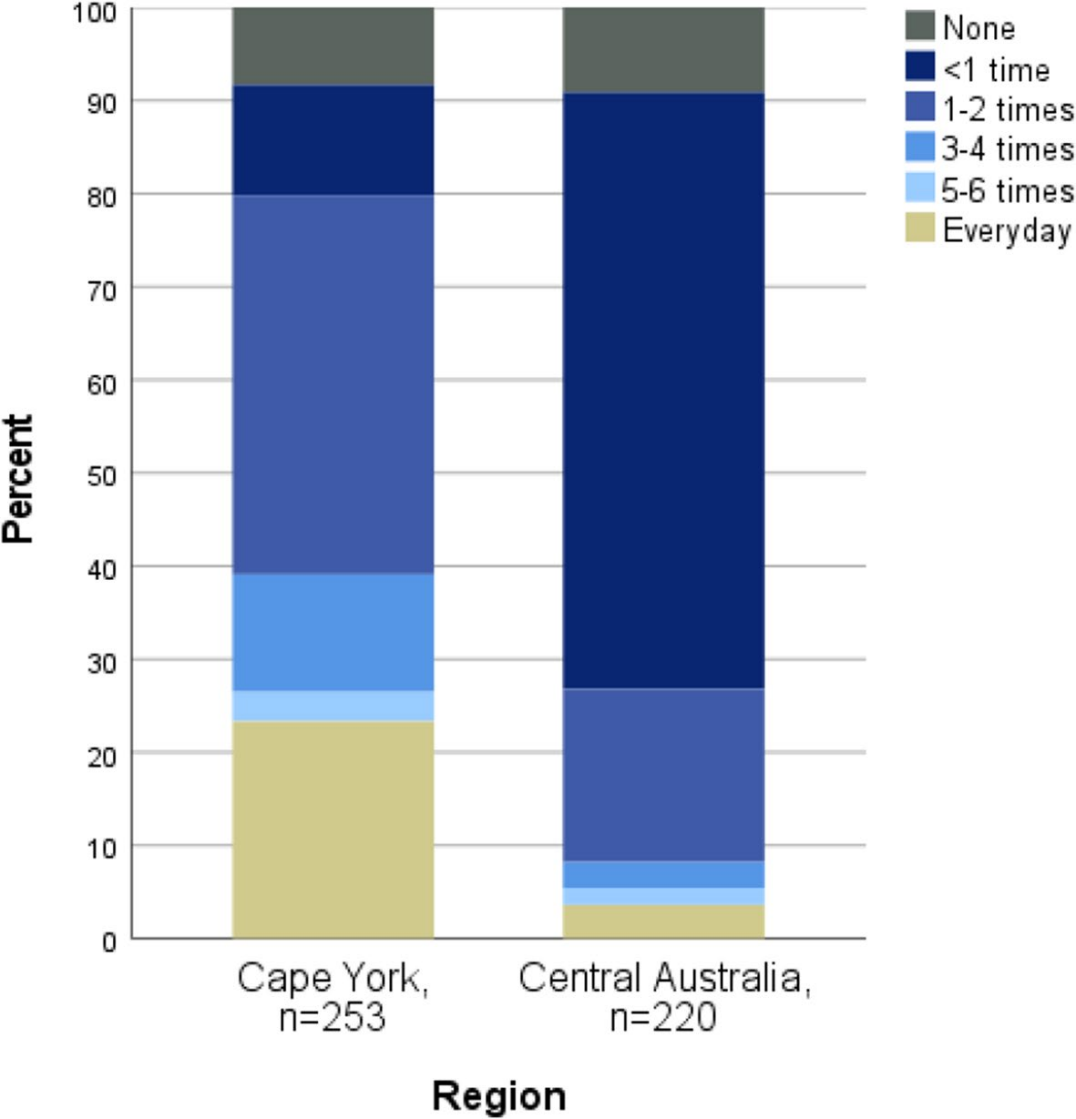
Frequency of traditional food consumption per week, by region

Traditional food intake frequency was higher in Cape York, where 39% of participants reported having traditional foods three or more times a week, compared to 8% of Central Australian participants (*p* < 0.001) (Fig. 1). Intake also varied by community within the regions, with participants in communities closest to the coast reporting the highest frequency of intake. This was reflected in the types of traditional foods reported consumed, including fish, crayfish, turtle, kangaroo, wallaby, and (introduced species) wild caught bullock and pig.

### Infant feeding

#### Breastfeeding

Of the 108 children aged up to two years, 58% were reported to be breastfed (> 6–12 months 66% breastfed, 1–2 years 56% breastfed). These breastfeeding rates persisted with 36% of children aged > 2–4 years breastfed. While breastfeeding rates in children under two years did not differ between regions (Cape York 53%, Central Australia 65%, *p* = 0.24), breastfeeding was more likely to continue beyond two years in Central Australia (Cape York 28%, Central Australia 46%, *p* = 0.03).

#### Minimum meal frequency

The overall MMF for all 90 children under two years with complete data was 94% (Breastfed children, *n* = 52, MMF = 92%; Non-breastfed children, *n* = 41, MMF = 98%). Children in Cape York were more likely to meet MMF than those living in Central Australia (98% compared with 88%, *p* = 0.03, respectively).

### Food security

**Fig. 2:**
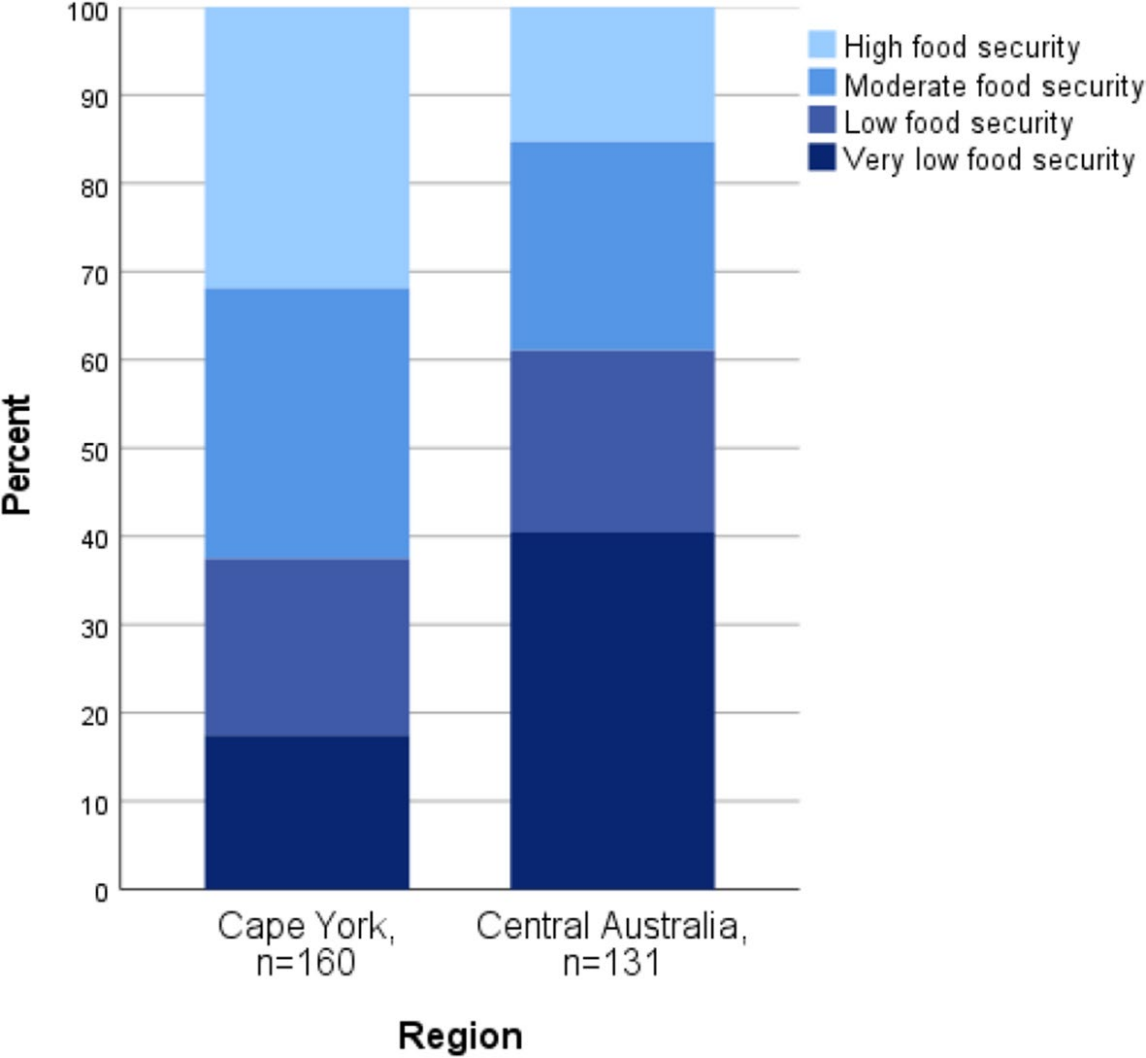
Food security status by region

Very few households experienced food security in all communities, with 76% of households classified as food insecure, including 28% experiencing very low food security (Fig. 2). Participants in Cape York were categorised as experiencing less severe food insecurity than Central Australian participants (*p* < 0.001).

### Interactions between diet quality, food security, frequency of traditional food intake and adult/child status

**Fig. 3:**
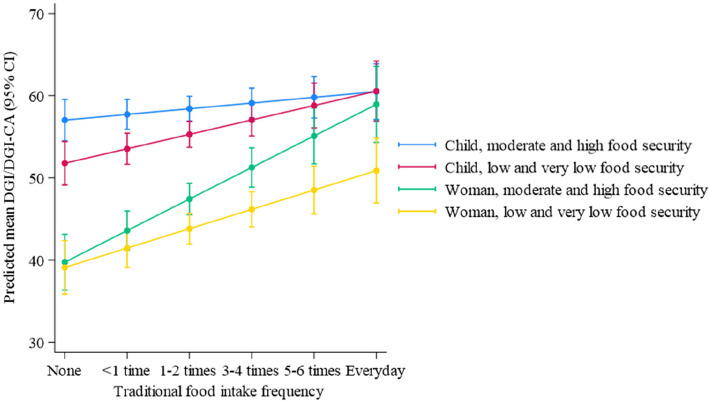
Predicted mean diet quality (DGI and DGI-CA score /100) from a model which included fixed effects for food security status, adult/child status, traditional food intake frequency and all two-way and three-way interactions, and random effects for families. Each point on the lines represents the predicted diet quality (y-axis, DGI and DGI-CA score /100) for a given traditional food intake frequency (x-axis), with the participants separated into groups for child/adult, and food security status. Positive line slope indicates a positive association between diet quality and traditional food consumption

For diet quality, the model indicated a significant three-way interaction between food security status, traditional food frequency and adult/child status (*p* = 0.005). The model was also fit for each region separately, with consistent results. The model predictions are shown in Fig. 3. For all groups (that is, women and children, and regardless of food security status), the upwards slope of the line indicates diet quality is positively associated with traditional food consumption (Fig. 3). Participants with low and very low food security, and the highest frequency of traditional food intake, had higher predicted diet quality than participants reporting moderate and high food security and the lowest frequency intake of traditional foods. That is, frequent traditional food intake reduces the impact of food insecurity on diet quality. Using women as an example in Fig. 3, the point at ‘Everyday’ for the yellow line (that is, the predicted diet quality for women with low and very low food security consuming traditional foods everyday, ~ 48 DGI points) is higher than the point at ‘None’ for the green line (the predicted diet quality for women with moderate and high food security consuming no traditional foods, ~ 40 DGI points).

**Fig. 4:**
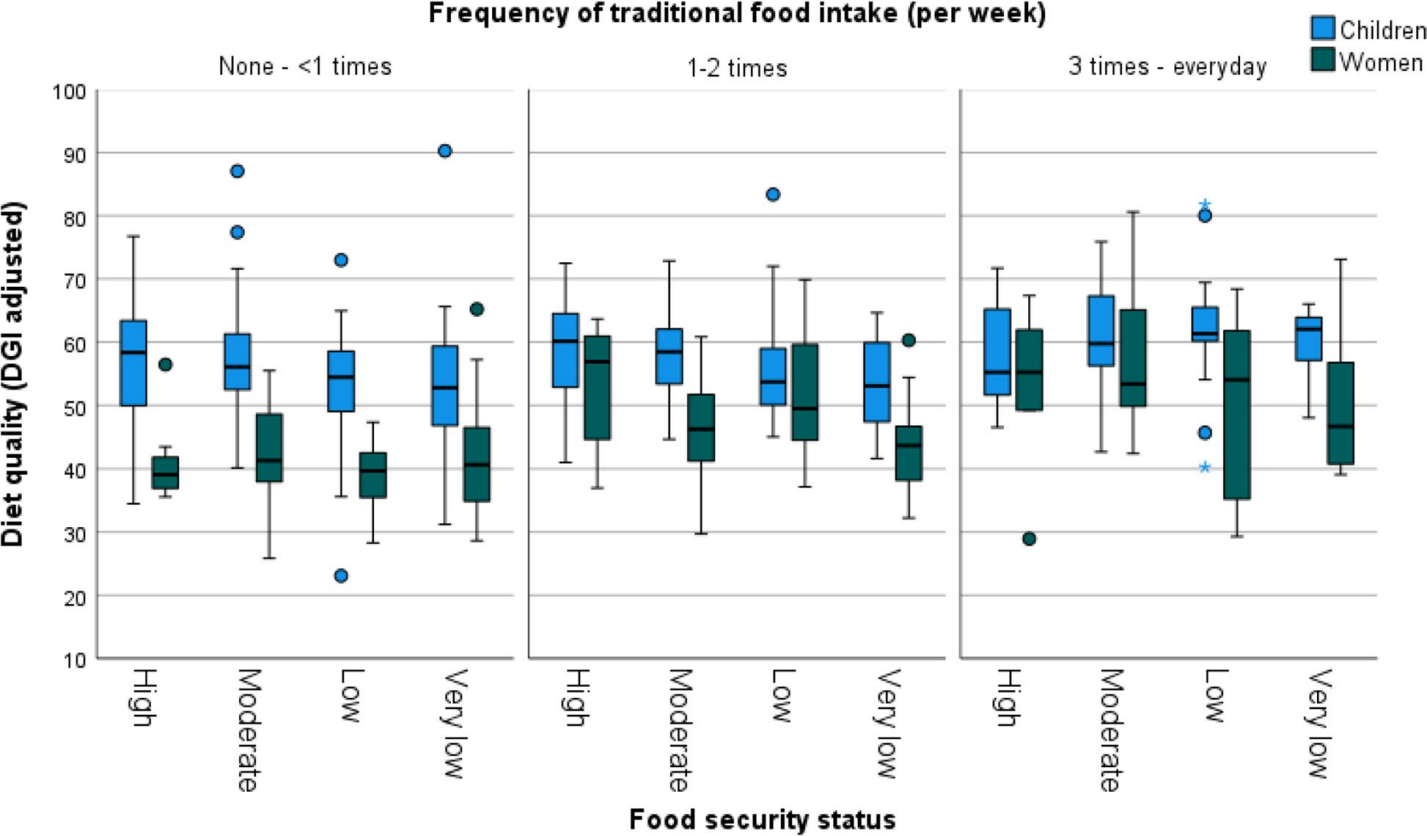
Diet quality, by frequency of traditional food intake and food security status, for women and children. Number of participants within each group, left to right: Children *n* = 25, *n* = 32, *n* = 22, *n* = 40, *n* = 25, *n* = 18, *n* = 17, *n* = 20, *n* = 14, *n* = 21, *n* = 15, *n* = 11; Women *n* = 7, *n* = 24, *n* = 11, *n* = 25, *n* = 13, *n* = 14, *n* = 10, *n* = 14, *n* = 6, *n* = 12, *n* = 10, *n* = 13. Box plot components: box, represents the IQR (25th-75th percentiles); box midline, median (50.^th^ percentile); whiskers, the furthest data points within 1.5 times the IQR of the quartiles; dot, mild outlier (between 1.5 and 3 times the IQR from the quartiles); asterisk, extreme outlier (more than 3 times the IQR away from the quartiles)

**Fig. 5:**
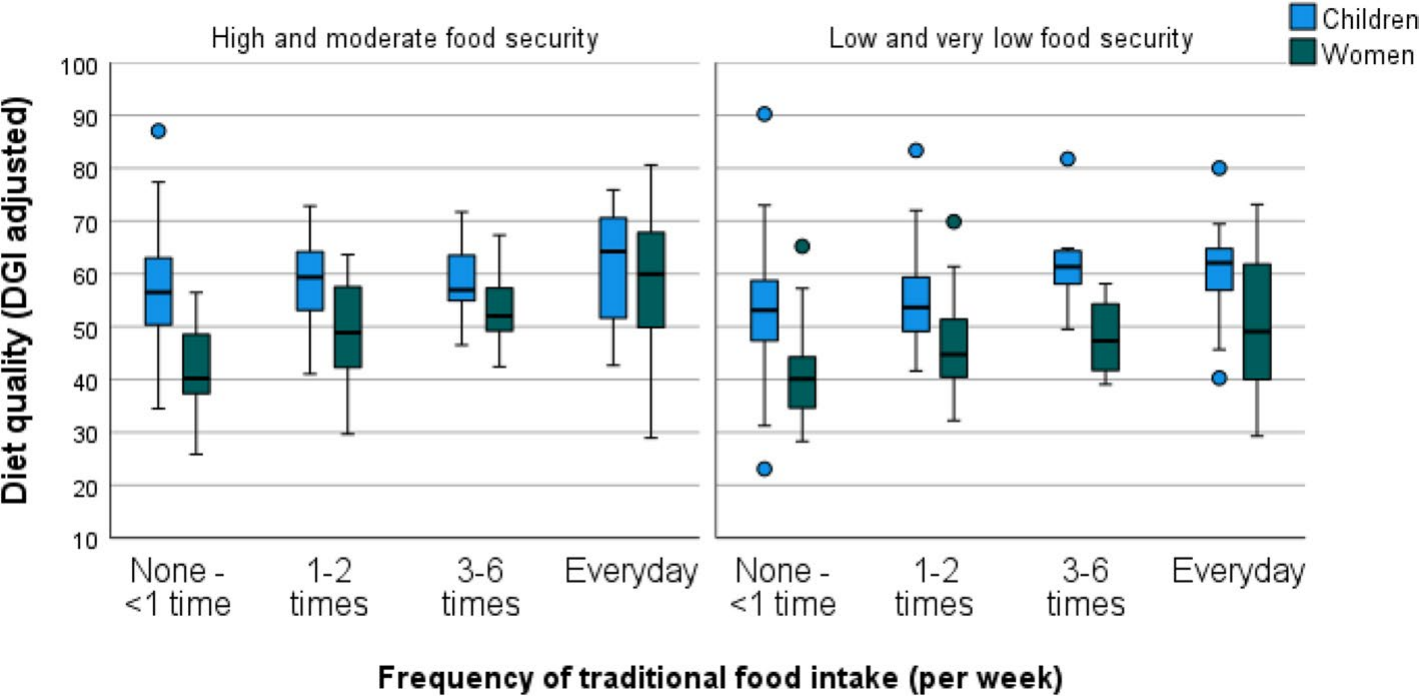
Diet quality by frequency of traditional food intake and food security status, for women and children. Number of participants within each group, left to right: Children *n* = 57, *n* = 43, *n* = 19, *n* = 16, *n* = 62, *n* = 37, *n* = 9, *n* = 17; Women *n* = 31, *n* = 27, *n* = 10, *n* = 8, *n* = 36, *n* = 24, *n* = 7, *n* = 16. Box plot components: box, represents the IQR (25th-75th percentiles); box midline, median (50.^th^ percentile); whiskers, the furthest data points within 1.5 times the IQR of the quartiles; dot, mild outlier (between 1.5 and 3 times the IQR from the quartiles); asterisk, extreme outlier (more than 3 times the IQR away from the quartiles)

Figures 4 and 5 provide further graphical demonstration of the interaction of the model factors using raw data. As anticipated, average diet quality was higher for children compared to women (see Figs. 4 and 5). Similarly, more frequent traditional food consumption was associated with higher diet quality in both groups (Figs. 4 and 5). However, this impact was stronger for women than children (Fig. 4), and this is also demonstrated by the steeper slopes of the lines representing women in Fig. 3 (green and yellow lines). Likewise, participants from more food secure households had higher diet quality relative to those consuming traditional foods with the same frequency from food insecure households, although there were some notable exceptions to this general trend. This trend was somewhat reversed in children with the most frequent consumption of traditional foods (Fig. 4 and women with the least frequent traditional food consumption had the lowest diet quality results, regardless of food security status (Fig. 4).

Therefore, as expected, frequent traditional food consumption attenuated the relationship between diet quality and adult/child status; that is, as traditional food intake frequency increased, the discrepancy in diet quality between women and children lessened (Fig. 4). This is also demonstrated in the converging of the model predicted lines in Fig. 3 at the highest traditional food intake frequency (Everyday). However, we anticipated that traditional food consumption would increase diet quality more strongly in food insecure households. While this was true for children as evidenced by the steeper slope of the red line compared to the blue in Fig. 3, we saw an opposite trend in women. That is, for women, more frequent traditional food consumption improved diet quality more strongly in households experiencing no, or less severe, food insecurity (Fig. 5), as evidenced by the steeper green line compared to the yellow in Fig. 3.

## Discussion

The dietary patterns and high rates of food insecurity reported by participants in this study demonstrate multiple systems failing Aboriginal and Torres Strait Islander peoples living in remote communities in Australia in the fundamental human right to have access to adequate food to meet their nutritional needs. Contrasting this overall picture are the findings showing community strength and resilience; highly prevalent and long duration breastfeeding, and in the coastal communities, frequent consumption of nutrient-dense traditional foods. The demonstrated interactions between diet quality, food security, traditional food intake and adult/child status accentuate the critical importance of supporting Aboriginal and Torres Strait Islander food systems as a mechanism for increasing diet quality and food security, particularly in pregnant and breastfeeding women.

The rates of household food insecurity reported in this study (76%) are higher than the nationally reported rate of 51% for Aboriginal and Torres Strait Islander households in remote communities (reported in 2022–23) [9], and vastly higher than for people in the Australian population (4%, reported in 2011–12) [63]. Our data supports prior evidence of a 76% prevalence shown for five remote Northern Territory communities [10]. Notably, unlike the national statistics, these higher rates are specifically among the households of pregnant or breastfeeding women and children under five years of age; both life stages of critical growth and development where optimising intake of key nutrients is essential for lifelong health [1, 2]. The interaction effects being stronger in women than children, coupled with the relatively superior diet quality of children, are supportive of qualitative research describing food security coping strategies which prioritise child feeding over adult feeding [31]. While this analysis shows these practices protect child diet quality in the context of household food insecurity, it is unacceptable that women, especially when they are pregnant and/or breastfeeding, face food system conditions that force them to choose between adequately feeding themselves or their children in remote Australia, a country with world-leading wealth [64].

The interaction findings suggest that, 1) more frequent traditional food consumption is associated with a reduction in the gap in maternal and child diet quality, 2) more frequent traditional food consumption is associated with higher diet quality in both women and children, reducing the negative impact of food insecurity on diet quality, and, 3) coastal communities with greater intake of traditional foods have lower rates of food insecurity. Collectively these findings provide evidence that supporting ongoing and increased access to traditional foods must be a central focus of all attempts to address the food security and health disparities experienced by Aboriginal and Torres Strait Islander people living in remote communities brought about by colonisation. This echoes community voice and other research describing the importance of, and threats to, ongoing access to traditional foods for Aboriginal and Torres Strait Islander peoples across Australia [7, 20, 23, 30, 31, 65]. This research demonstrates that while both geographic areas experience food insecurity, more support is needed to facilitate access in Central Australian desert communities in particular, where ethnographic research has also reported low traditional food intakes [21]. This research provides further evidence for the importance of this from a nutritional perspective, but the importance of access to traditional foods in fostering social and cultural connection and identity, and therefore health and spiritual wellbeing, cannot be overstated [4, 5, 7, 66].

The diet quality outcomes reported for the youngest children in this study (< 2 years) were similar, if not closer to recommendations, than reports from other remote community populations using comparable measures [17, 58], and demonstrated similar patterns of consumption overall to other studies [18]. They are also similar to patterns reported from a nationally representative population although with many more children meeting recommended meat intakes in our analysis [67], likely due to traditional food consumption. Additionally, the breastfeeding maintenance rates are similar to the nation leading results reported from other remote cohorts [17, 18, 68], and higher than those reported for rural and urban Aboriginal and Torres Strait Islander populations [16, 68–70]. Smithers, Hedges [12] and Onifade, Pringle [13] report higher intakes of vegetables and fruit in children, as do studies reporting maternal diets [15, 71]. Given the majority of participants in these studies were not living remotely, this likely reflects better access and quality of produce available in non-remote locations, as reported qualitatively [27].

Nonetheless, our findings support reports of dietary intake with Aboriginal and Torres Strait Islander women from non-remote locations showing patterns of low food group and/or nutrient intakes and low diet quality [14, 15, 71, 72]. Additionally, while meat intakes on average met requirements for women, it has been noted that the recommended meat intakes alone may not be sufficient to meet iron requirements in pregnancy [73], which is particularly relevant in the remote community setting where iron deficiency anaemia continues to be prevalent [74]. Our findings also support patterns of SSB consumption reported in extant literature, with low consumption in infants 6–12 months, and consumption increasing with increasing age [18], to high rates of consumption in older children and adults [75–77]. Given the role maternal nutrition plays in setting lifetime metabolic and disease trajectories intergenerationally [1–3], these reported intakes demand action. The causal links between the social determinants of health experienced by Aboriginal and Torres Strait Islander peoples and the dietary patterns reported here are well documented [21, 23, 78, 79]. These results show little progress has been made towards improvements in food insecurity and therefore its determinants [24–26, 78] which are crucial to addressing the issues of diet quality reported here, which underpin the disproportionate burden of diet-related chronic disease experienced by Aboriginal and Torres Strait Islander peoples living remotely [4, 80]. Efforts to increase access to traditional foods should be prioritised alongside strategies to address the broader social and structural determinants of food security, in order to improve food security and diet quality outcomes [29, 31].

A strength of this work was the involvement of community researchers and leaders in project implementation, but importantly in interpretation of the findings. Extensive discussion of preliminary results was conducted in all communities, providing confidence in the accuracy of these results in reflecting the food security and diet quality picture for each community. This was particularly important given data collectors reported participant discomfort while completing the food security survey, especially questions relating to child food security, which undoubtedly reflects historical and current trauma associated with the disproportionately high removal of Aboriginal and Torres Strait Islander children [81]. While this study focused on the diet quality of women and young children as primary participants of the study it did not examine the diet quality of other family members such as men, older children and adolescents, or older adults, which may provide further context within families and communities. Additionally, some participants reported difficulty answering questions due to the economic focus of the survey not capturing the food procurement practices in community that do not require money, such as sharing foods and wild harvesting [31]. When comparing the tool to concepts included in the Aboriginal definition of food security cited earlier, it is clear that the tool falls short in capturing the whole food environment described, particularly access to traditional foods [19]. This was contrasted by the reported relative participant satisfaction with the MRSDAT, a tool developed with and for Aboriginal and Torres Strait Islander populations [52]. With the ongoing need to reliably assess and monitor food security to determine progress towards the structural changes required to reduce health inequities, investment into the development of Aboriginal and Torres Strait Islander specific tools, co-designed with Aboriginal and Torres Strait Islander leadership, is needed. Additionally, while foods and dietary patterns considered health promoting by Aboriginal and Torres Strait Islander peoples themselves are not specifically incorporated in the dietary patterns reported as ideal in the ADGs, (although recommendations to enjoy traditional foods whenever possible and use store foods which are most like traditional food are [57]), a comparison of reported diets against the ADGs is appropriate as they are the dietary reference recommended by the National Health and Medical Research Council for all Australians, including Aboriginal and Torres Strait Islander peoples [82].

As only one question was asked about breastfeeding our ability to describe initiation, frequency or exclusivity of breastfeeding is limited. Similarly, we did not provide strict definitions for breastfeeding or traditional food for participants, but elected to enable participants to interpret their own meaning of these terms, which did limit comparability of our data with other studies. However, further strengths of the study include the involvement of a large proportion of the eligible population of eight communities, across two geographical regions, using multiple tools, enabling a comprehensive picture of dietary intake in a large part of remote Australia. That this was achieved during severe travel restrictions during the COVID-19 pandemic is also a great achievement. This large and geographically diverse dataset also enabled the analysis of the relationships between diet quality, food security, traditional food consumption, and adult/child status which is the first statistical demonstration of these interactions in Aboriginal and Torres Strait Islander remote community populations. The model incorporated traditional food intake as a linear variable measuring frequency only, and further exploration incorporating types of foods would be a valuable extension of this, given the diversity of traditional foods consumed in remote communities across Australia.

## Conclusions

This research shows practices to protect and support the diet quality of Aboriginal and Torres Strait Islander children, such as long breastfeeding maintenance, are enacted within remote communities. The novel statistical demonstration of the importance of traditional food consumption in reducing the impact of food insecurity on diet quality provides further support for Aboriginal and Torres Strait Islander food systems to be at the centre of comprehensive efforts to address food insecurity. As such, we strongly support the community-identified recommendations to support traditional food consumption identified in the National Strategy for Food Security in Remote Aboriginal and Torres Strait Islander Communities, these being: the development of guidance around rights and access to traditional resources, ongoing funding for traditional food collection and community-led industries, and development of an Aboriginal and Torres Strait Islander workforce that integrates traditional food knowledge into health education [83]. This research also highlights the impact of the regional disparities in intake of traditional foods, with urgent policy changes needed to address this. Research and policy must focus on Indigenous-led solutions to the challenges posed by remote food systems that are currently failing Aboriginal and Torres Strait Islander peoples in their right to adequate food.

## Data Availability

No datasets were generated or analysed during the current study.

## Abbreviations

ADG(s): Australian Dietary Guidelines
DGI: Dietary Guideline Index score (diet quality score) for adults
DGI-CA: Dietary Guideline Index (diet quality score) for children and adolescents
MMF: Minimum Meal Frequency
MRSDAT: Menzies Remote Short-item Dietary Assessment Tool
SSB: Sugar Sweetened Beverages
